# 

**Published:** 1898-10

**Authors:** 


					﻿Vol. XVIII.
OCTOBER, 1898.
NO. 10?
THjE
pOMEDPATHKJ-pHY^lClAjI,
A Monthly Journal of Homoeopathic Materia Medica
and Clinical Medicine.
“ He it a freeman whom truth makes free, and all are slaves beside."
"Seek the Truth: come whence it may, cost what it will.’’
BDITBD AND PUBLISHED BY
WALTER M. JAMES, M. D.
PHILADELPHIA:
ZFSTo. 1231 LOCUST STREET.
W Yearly Subscription, $2.SO, invariably tn adnwtoe. Single niunbvt, 2S cte.
Entered at the Philadelphia Poit-Office as Second-Cl as« Mail Matter.
				

